# A *Bordetella pertussis* MgtC homolog plays a role in the intracellular survival

**DOI:** 10.1371/journal.pone.0203204

**Published:** 2018-08-30

**Authors:** Juan Hilario Cafiero, Yanina Andrea Lamberti, Kristin Surmann, Branislav Vecerek, Maria Eugenia Rodriguez

**Affiliations:** 1 CINDEFI (UNLP CONICET La Plata), Facultad de Ciencias Exactas, Universidad Nacional de La Plata, La Plata, Argentina; 2 Interfaculty Institute for Genetics and Functional Genomics, Department of Functional Genomics, University Medicine Greifswald, Greifswald, Germany; 3 Laboratory of Molecular Biology of Bacterial Pathogens, Institute of Microbiology of the ASCR, v.v.i., Prague, Czech Republic; 4 Laboratory of post-transcriptional control of gene expression, Institute of Microbiology of the ASCR, v.v.i., Prague, Czech Republic; Centre National de la Recherche Scientifique, FRANCE

## Abstract

*Bordetella pertussis*, the causative agent of whooping cough, has the capability to survive inside the host cells. This process requires efficient adaptation of the pathogen to the intracellular environment and the associated stress. Among the proteins produced by the intracellular *B*. *pertussis* we identified a protein (BP0414) that shares homology with MgtC, a protein which was previously shown to be involved in the intracellular survival of other pathogens. To explore if BP0414 plays a role in *B*. *pertussis* intracellular survival a mutant strain defective in the production of this protein was constructed. Using standard *in vitro* growth conditions we found that BP0414 is required for *B*. *pertussis* growth under low magnesium availability or low pH, two environmental conditions that this pathogen might face within the host cell. Intracellular survival studies showed that MgtC is indeed involved in *B*. *pertussis* viability inside the macrophages. The use of bafilomycin A1, which inhibits phagosome acidification, abolished the survival defect of the *mgtC* deficient mutant strain suggesting that in intracellular *B*. *pertussis* the role of MgtC protein is mainly related to the bacterial adaptation to the acidic conditions found inside the of phagosomes. Overall, this work provides an insight into the importance of MgtC in *B*. *pertussis* pathogenesis and its contribution to bacterial survival within immune cells.

## Introduction

Whooping cough is a highly contagious infectious disease caused by *Bordetella pertussis*. It is a life threatening disease that persists as a major cause of infant morbidity and mortality worldwide despite years of vaccination [1]. Previous studies have shown that *B*. *pertussis*, historically considered as an extracellular pathogen, once internalized into different host cells, including neutrophils, macrophages, and epithelial cells, has the capacity to survive inside these cells [2–4]. Moreover, in the absence of opsonic antibodies a significant fraction of macrophage-phagocytosed *B*. *pertussis* has the capacity to prevent phagosome-lysosome fusion, eventually replicating in compartments with early endosomal characteristics [3]. This ability to survive intracellularly might contribute to vaccine failure and the persistence of this pathogen within the host [5–7]. In order to survive within the intracellular environment pathogens not only need to evade the cellular killing activity of the host cell but also have to adapt their metabolism to the available nutrients and to environmental conditions inside the cellular compartments. We previously published a global proteomic study showing the evolution of *B*. *pertussis* proteome during the intracellular infection of macrophages [8]. Among the proteins expressed by the intracellular *B*. *pertussis* a homolog of MgtC, BP0414, was identified. MgtC is a virulence factor that has been horizontally acquired by many pathogens during evolution [9, 10]. Several studies indicate that MgtC is a key subversion factor that contributes to intramacrophage adaptation and intracellular survival [9]. Despite the differences in pathogenic traits among the microorganisms that produce MgtC, this virulence factor has a similar function in all of them, namely, the promotion of growth in acidic environments and under magnesium starvation [9].

Although MgtC was first considered a magnesium transporter [11], it has been shown that this protein is actually involved in ATP homeostasis by inhibiting the bacterial F_1_F_o_ ATP synthase when bacteria encounter adverse environmental conditions, such as a decrease in the pH or in the Mg^2+^ concentration. This inhibition is crucial not only to avoid the production of supra-physiological levels of ATP during growth under acidic pH, but also to restore intracellular magnesium levels [12]. Magnesium is a divalent cation involved in several cellular processes, such as the stabilization of membranes and ribosomes, nucleic acids neutralization, and it is a cofactor in a variety of enzymatic reactions [13]. Both magnesium and ATP homeostasis are critical for bacterial survival and replication.

As mentioned above, *B*. *pertussis* resides and survives inside human macrophages in compartments with early endosomal characteristics in which the environment is mildly acidic. Under these environmental conditions MgtC might play a relevant role in bacterial viability. In this study, we investigated the contribution of BP0414, hereafter called MgtC, to the development of intracellular infections.

## Materials and methods

### Bacterial strains and growth conditions

Bacterial strains and plasmids used in this study are described in Table 1. *Escherichia coli* strains were cultured on Luria-Bertani (LB) agar at 37°C or in LB broth at 37°C under shaking conditions. *Bordetella pertussis* Tohama and its derivatives were grown on Bordet Gengou agar (BGA) (BD Difco, New Jersey, USA) plates supplemented with 15% (v/v) defibrinated sheep blood (Laboratorio Argentino, Caseros, Argentina) (bBGA) at 37°C. Animal handling and all procedures were in compliance with the Argentinean animal protection Law 14346. For infection experiments *B*. *pertussis* strains were grown on bBGA, and subcultured in Stainer-Scholte (SS) liquid medium [14] for 16 h at 37°C under shaking conditions.

**Table 1:** Strains and plasmids used in this study.

Strain or plasmid	Description or phenotype^a^	Source or reference
**Strains**		
***E*. *coli* DH5𝛂**	General cloning host	Stratagene
***E*. *coli* SM10(λPir)**	Donor strain for conjugation	[15]
***B*. *pertussis* Tohama**	wild-type genotype, Cex^R^	Pasteur Institute, Paris, France
***B*. *pertussis* Δ*mgtC***	Tohama derivative, *mgtC* mutant strain	This study
***B*. *pertussis* Δ*mgtC* pBBR-*mgtC***	*mgtC* mutant strain harbouring the complementation plasmid pBBR1MCS-*mgtC*	This study
***B*. *pertussis* Δ*mgtC* pBBR**	*mgtC* mutant strain harbouring the empty plasmid pBBR1MCS	This study
**Plasmids**		
**pSS4245**	Allelic exchange vector, Km^R^, Amp^R^, Sm^R^	[16]
**pSS4245Δ*mgtC***	pSS4245 derivative, *mgtC* deletion plasmid	This study
**pBBR1MCS**	Broad-host-range plasmid, Cm^R^	[17]
**pBBR1MCS-*mgtC***	*mgtC* complementation plasmid, pBBR1MCS derivative plasmid containing the *mgtC* locus	This study

^a^Cex^R^, cephalexin resistant;, Km^R^, kanamycin resistance; Amp^R^, ampicillin resistance; Sm^R^, streptomycin resistance; Cm^R^, chloramphenicol resistance

For growth under magnesium limiting conditions, *B*. *pertussis* strains were grown on bBGA and subcultured in SS liquid modified medium containing 10 μM of MgCl_2_ (SS-Mg) to an optical density at 650 nm (OD) of 0.2. The MgCl_2_ concentration in SS-Mg was chosen because it has been used to test the involvement of MgtC for growth in Mg^2+^-deprived media in several bacteria [18]. SS-Mg cultures were grown at 37°C under shaking conditions for 16 h. Bacterial cells were harvested by centrifugation (10,000 x *g* for 15 min at room temperature), washed with sterile Mg^2+^ free saline solution, and diluted to an estimated concentration of 2 x 10^9^ CFU/mL. Equal volumes of bacterial cell suspensions were used to inoculate fresh SS (500 μM of MgCl_2_) or SS-Mg to an OD of 0.2. RNA samples for RT-qPCR studies were isolated from bacterial samples taken in the exponential growth phase.

For bacterial growth under different pH defined conditions, *B*. *pertussis* strains were grown on bBGA and subcultured in SS medium to an estimated concentration of 2 × 10^8^ CFU/mL. Cultures were grown at 37°C under shaking conditions for 16 h. Bacterial cells were harvested by centrifugation (10,000 x *g* for 15 min at room temperature), washed with sterile saline solution, and diluted to an estimated concentration of 2 x 10^9^ CFU/mL. Equal volumes of bacterial cell suspensions were used to inoculate SS modified media in which Tris buffer was replaced by 50 mM of 2-(N-Morpholino)-ethanesulfonic acid (MES) (Sigma-Aldrich, Saint Louis, USA) (SS-MES) and the pH was adjusted to 7.6 or 6.0. Bacterial cultures in SS-MES at pH 7.6 or 6.0 (initial OD: 0.2) were performed at 37°C under shaking conditions. RNA samples for RT-qPCR studies were obtained from bacterial samples taken in the exponential growth phase.

### Construction of the Δ*mgtC* mutant strain

*B*. *pertussis mgtC* deletion mutant strain was obtained by homologous recombination using the allelic exchange vector pSS4245 as described in [16]. Briefly, two DNA fragments of approximately 750 bp corresponding to either the 5’ or the 3’ flanking region of the *mgtC* gene were generated using PCR as follows. The upstream region of *mgtC* was amplified using forward primer 5’-CTGCGGCCGCCCTTCGGCAATCAGCGTCATG-3’ containing a *Not*I site (underlined) and reverse primer 5’-GAACTAGT**CATG**GCTGCGTGCTCCCGG-3’ containing a *Spe*I site (underlined) and the ATG initiation codon of the *mgtC* gene (bold). Similarly, the downstream region of *mgtC* was amplified using forward primer 5’-CTACTAGTAAGCGGGTCGTCGCGCAGC-3’ containing a *Spe*I site (underlined) and reverse primer 5’-GAGGATCCCCTGGAAATCCTGGTCGATC-3’ containing a *Bam*HI site (underlined). The first PCR product was cleaved with *Not*I and *Spe*I and the second PCR product with *Spe*I and *Bam*HI. Both cleaved products were ligated with the allelic exchange plasmid pSS4245 [16] previously cleaved with *Not*I and *Bam*HI. The resulting plasmid, pSS4245Δ*mgtC*, contains an insert with the start codon and the last ten codons of *mgtC* separated by a *Spe*I restriction site along with the flanking regions, creating a markerless in-frame *mgtC* deletion. The pSS4245Δ*mgtC* was transformed into the *E*. *coli* SM10 λpir strain (donor strain) and transferred to *B*. *pertussis* Tohama I (recipient strain) by conjugation, as described elsewhere [16]. As a result of two subsequent recombination events, the strain carrying a chromosomal in-frame deletion of the *mgtC* gene was obtained. The deletion of the *mgtC* gene and the sequences of its 5’ and 3’ adjacent regions in the mutant strain were confirmed by DNA sequencing (S1 Fig). Control experiments in which pSS4245 empty plasmid was transferred to *B*. *pertussis* yielded no colonies in selective medium.

### Complementation of the Δ*mgtC* mutant strain

To obtain a strain in which the *mgtC* deletion would be complemented, a plasmid pBBR1MCS-*mgtC* carrying the *mgtC* gene of *B*. *pertussis* Tohama strain including its own promoter and terminator sequences was constructed as follows. A fragment containing the *mgtC* gene was amplified by PCR using the forward primer 5’- CTACTAGTCGGCGTCTCCAACGCAGGC-3’ containing a *Spe*I site (underlined) and the reverse primer 5’-GAAAGCTTCCGAGCAGATCATCGCTGCC-3’ containing a *Hin*dIII site (underlined). The PCR product was cleaved with *Spe*I and *Hin*dIII and inserted into the corresponding sites of the cloning vector pBBR1MCS [17]. The resulting plasmid, pBBR1MCS-*mgtC*, was transferred to the *B*. *pertussis* Δ*mgtC* strain by conjugation. The *B*. *pertussis* Δ*mgtC* strain was also transformed with the empty pBBR1MCS vector as a control. *B*. *pertussis* transconjugants were selected in bBGA plates supplemented with chloramphenicol 20 μg/mL and cephalexin 100 μg/mL. All plasmid constructs were confirmed by DNA sequence analysis.

### Cells culture conditions and infection

The human monocytic cell line THP-1 (ATCC TIB-202, Rockville, MD) was cultured in RPMI 1640 (Gibco, Grand Island, NY, USA) supplemented with 10% (v/v) of fetal bovine serum (Gibco) (RPMI-FBS) at 37°C in a humidified 5% (v/v) CO_2_ air atmosphere. Infection assays were performed as described before [8] with minor modifications. Briefly, 4 × 10^5^ THP-1 cells/mL were suspended in RPMI-FBS containing 100 nM of phorbol-12-myristate-13-acetate (PMA) (Sigma-Aldrich) and plated for differentiation into macrophages. After 24 h, monolayers were washed with sterile phosphate-buffered saline (PBS) and infected with *B*. *pertussis* in RPMI 1640 plus 0.2% (w/v) bovine serum albumin (BSA) (Sigma-Aldrich) at a multiplicity of infection (MOI) of 100 bacteria per cell. To facilitate bacterial interaction with THP-1 cells, plates were centrifuged for 5 min at 640 × *g* at room temperature. After 2 h at 37°C in 5% (v/v) CO_2_, infected cells were washed with PBS to remove non-adherent bacteria and fresh RPMI-FBS medium containing 100 μg/mL polymyxin B sulfate (Sigma-Aldrich), an antibiotic that cannot penetrate mammalian cells [19], was added to kill the remaining extracellular bacteria. After 1 h at 37°C, the cells were washed with PBS and either lysed with sterile water (3 h post-infection) or incubated with fresh RPMI-FBS medium plus polymyxin B (5 μg/mL) for 21 h or 45 h (24 h and 48 h post-infection, respectively). The number of CFUs in the culture supernatants was examined and no viable bacteria were detected at any time post-infection. At the selected time points post-infection cell samples were washed with PBS and lysed with sterile water. Serial dilutions of the lysates were rapidly plated onto bBGA plates and incubated at 37°C for 5 days to enumerate CFUs in each well. Viable intracellular bacteria were expressed as the number of viable bacteria per mL. No significant differences in bacterial sensitivity to distilled water were detected among the *B*. *pertussis* strains used in this study. Control experiments to assess the efficacy of antibiotic bactericidal activity were performed in parallel. Briefly, samples of 4 × 10^7^ bacteria, the number of bacteria per well used for the infection experiments, were incubated with 100 μg/mL polymyxin B for 1 h at 37°C and plated on bBGA. This incubation resulted in a 99.9999% decrease in CFUs. No significant differences in bacterial sensitivity to the antibiotics were detected among the *B*. *pertussis* strains used in this study. The viability of infected THP-1 cells was determined at the different times after infection with trypan blue. No significant cell death was observed in any of the different infection assays performed in this study.

### Confocal microscopy analysis

The number of extracellular and intracellular bacteria 2 hours post-infection was determined by immunofluorescence staining as described elsewhere [8]. Briefly, samples of infected THP-1 macrophages were fixed with 3% (w/v) paraformaldehyde (PFA) and surface-bound bacteria were detected by incubation with rabbit anti-*B*. *pertussis* serum, obtained as described elsewhere [20], for 1 h at 4°C, followed by the incubation with Cy3-conjugated goat F(ab′)_2_ fragments of anti-rabbit IgG (Jackson ImmunoResearch, West Grove, PA) for another 1 h at 4°C. In order to determine the number of intracellular bacteria, the cells were permeabilized by incubation with PBS containing 0.1% (w/v) saponin (Sigma-Aldrich) and 0.2% (w/v) BSA for 30 min at room temperature, followed by incubation for 1 h at room temperature with rabbit anti-*B*. *pertussis* serum in the presence of 0.1% (w/v) saponin and 0.2% (w/v) BSA. After three washing steps the cells were further incubated with FITC-conjugated F(ab′)_2_ fragments of goat anti-rabbit IgG (Jackson ImmunoResearch, West Grove, PA) in the presence of 0.1% (w/v) saponin and 0.2% (w/v) BSA for 1 h at room temperature. After washing, the samples were analyzed by fluorescence microscopy under a confocal laser scanning microscope (model TCS SP5; Leica, Germany). Extracellular bacteria were seen double labeled (red and green) while intracellular bacteria were labeled green only allowing the discrimination between attached and phagocytosed bacteria. Bacterial phagocytosis was evaluated by examination of at least 50 eukaryotic cells from each condition tested.

Colocalization studies were performed as described before [3] with minor modifications. Briefly, THP-1 cells differentiated into macrophages by PMA were infected with *B*. *pertussis* (MOI: 100) as described before. After 2 h of incubation at 37°C with 5% (v/v) CO_2_, non-adherent bacteria were removed by three washing steps with PBS. Extracellular bacteria were then killed with polymyxin B (100 μg/mL) for 1 h and further incubated either with 75 nM LysoTracker DND-99 (Molecular Probes, Eugene, OR) (5 min at 37°C), followed by fixation with paraformaldehyde, or with 5 μg/mL polymyxin B for colocalization studies at later time points. At 24 and 48 h post-infection, macrophage samples were incubated with LysoTracker prior to fixation with paraformaldehyde. LysoTracker-treated cells were washed twice with PBS and incubated for 10 min at room temperature with PBS containing 50 mM NH_4_Cl. After three washing steps, THP-1 cells were incubated for 30 min with PBS containing 0.1% (w/v) saponin and 0.2% (w/v) BSA. Next, the cells were incubated for 1 h at 4°C with rabbit anti-*B*. *pertussis* serum in the presence of 0.1% (w/v) saponin and 0.2% (w/v) BSA. After three washing steps, THP-1 cells were incubated (1 h) with FITC-conjugated F(ab´)_2_ fragments of goat anti-rabbit IgG (Jackson ImmunoResearch). Microscopic analyses were performed using a confocal laser scanning microscope (Leica TCS SP5). The percentage of phagosomes containing bacteria that colocalized with LysoTracker was calculated by analyzing at least 50 phagosomes per sample.

### Macrophage treatment with vacuolar pH-neutralizing reagent

Neutralization of vacuolar pH was performed by incubating THP-1 cells with bafilomycin A1 (BAF) (Sigma-Aldrich), an inhibitor of vacuolar v-ATPase [21]. To this end, one hour before infection, 50 nM BAF dissolved in DMSO was added to PMA differentiated THP-1 cells and maintained during the assay. In control experiments, the effect of BAF on phagosomal pH was confirmed by incubating THP-1 macrophages treated with or without BAF with LysoTracker. No LysoTracker-positive organelles were observed in BAF-treated macrophages, as assessed by confocal microscopy (S2 Fig). There was no toxic effect of BAF on *B*. *pertussis* or macrophages, as determined by CFU counts and trypan blue dye exclusion, respectively.

### Sample preparation and data acquisition for single reaction monitoring (SRM) mass spectrometry

Intracellular *B*. *pertussis* was isolated at 3 h and 48 h post-infection from THP-1 infected cells as described before [8]. Bacteria growing in RPMI 1640 BSA 0.2% (w/v) during 3 h at 37°C in a humidified 5% (v/v) CO_2_ air atmosphere were used as a control (named as extracellular bacteria). Isolated intracellular bacteria and the control bacteria were further processed for SRM analysis as described before [8]. In brief, samples were dissolved in aqueous 8 M urea / 2 M thiourea (UT) buffer and proteins were solubilized by shaking. Samples were centrifuged at 20,000 × *g* at 20°C for 1 h and total protein concentration was measured in the supernatant using a Bradford assay (Bio-Rad, München, Germany) with BSA as reference. Four μg of proteins in UT-buffer per sample were further diluted to a final concentration of 1 M urea / 0.25 M thiourea in 20 mM aqueous ammonium bicarbonate (ABC), reduced with 2.5 mM dithiothreitol (DTT) for 1 h at 60°C, alkylated with 10 mM iodoacetamide in 20 mM ABC for 30 min at 37°C, and proteolytically digested with trypsin (protein to trypsin ratio 25:1; Promega, Madison, WI, USA) overnight at 37°C. Digestion was stopped by addition of trifluoroacetic acid (TFA, Merck, Darmstadt, Germany) to a final concentration of 0.1% (v/v). Peptides were purified and desalted using C18-ZipTip columns (MerckMillipore, Billerica, MA, USA). Peptides were then re-suspended in buffer containing 2% (v/v) ACN and 0.1% (v/v) acetic acid in HPLC grade water (J. T. Baker, Center Valley, PA, USA). SRM analysis was performed on two independent biological replicates including two technical replicates each using a TSQ Vantage (Thermo Scientific, Bremen, Germany) after separation of peptides by a nanoAcquity UPLC (Waters Corporation, Manchester, UK) at a flow rate of 300 nL/min employing a nonlinear gradient ranging from 5% to 90% ACN (v/v) in 33 min (0 min-5% ACN / 3–10 / 26–35 / 32–60 / 33–90 / 38–90 / 39–0 / 42–0). Precursor fragmentation was performed using collision induced dissociation according to factory defaults. Settings for final SRM analyses were adjusted to a MS resolution for MS1 of 0.7 full width at half maximum (FWHM), cycle time of 1 s/cycle and a declustering voltage of 1 V.

Setup and validation of transitions as well as peak area calculation were established using the program Skyline v3.5 [22]. Being a small protein (about 18 kDa), peptide sequence of BP0414 did not provide more than one putative proteotypic peptide, LGNEGEIR, which fulfils all the properties for exact SRM analysis [23] and was confirmed to be a unique amino acid sequence among the whole *B*. *pertussis* Tohama I genome controlled by sequence comparison using NCBI (https://www.ncbi.nlm.nih.gov/). Therefore, BP0414 was analysed with one peptide. In addition, peptides of two housekeeping proteins [MaeB (BP1120) and GdhA (BP1857)] were used as reference for normalization as done before [8]. The final transition list is provided as S1 Table. Identification of peptide LGNEGEIR in the different samples was validated by retention time and transition pattern of the peak. The final quantification between 3 h post-infection, 48 h post-infection, and the control was carried out on protein level by averaging the related peptide area ratios. In order to normalize the data, the average areas of the two housekeeping proteins were used and the final results were given as ratios related to the control as described before [8]. In our previous study [8], likewise prepared samples were analysed by shotgun mass spectrometry. These data are already published at the ProteomeXchange Consortium [24, 25] via the PRIDE partner repository with the identifier PXD002997 and are referred to in this article.

### RNA extraction and quantitative RT-PCR (RT-qPCR)

Total RNA was prepared from bacteria obtained at exponential growth phase with the RNeasy Mini Kit according to the manufacturer’s instructions (Qiagen, Valencia, CA, USA), including a treatment with DNase I (Promega, Madison, WI). RNA quality was assessed both by agarose gel electrophoresis and photometrically by NanoDrop2000 (Thermo Scientific). Synthesis of cDNA was performed with the M-MLV Reverse Transcriptase (Promega) following the manufacturer's instructions. For cDNA synthesis, 1 μg of a total RNA and 10 μM random hexamers (Qiagen) were used. A control reaction without the M-MLV Reverse Transcriptase was included to confirm the absence of genomic DNA in RNA samples. RT-qPCR analyses were performed on an Mx3000P qPCR System (Stratagene, San Diego, USA) with the SYBR GreenPCR Master Mix (Roche, Mannheim, Germany) and 0.5 μM of each forward and reverse primer. Primer sequences are listed in Table 2. PCR runs comprised a 10 min preincubation at 95°C, followed by 40 cycles of a two-steps PCR consisting of a denaturing step at 94°C for 15 sec and a combined annealing and extension phase at 60°C for 60 sec. The resulting amplicons were examined by melting peaks. The relative expression level of each gene was calculated by the threshold cycle (ΔΔCt) method [26]. The *recA* gene was used as a reference gene for normalization of gene expression data.

**Table 2:** Primers used for RT-qPCR.

Gene	Locus tag	Forward primer	Reverse primer
***mgtC***	BP0414	ATCAAGCTGGGCAACGAG	GCGATGCGCTTTTCCAG
***recA***	BP2546	GACGACAAAACCAGCAAGG	CGTAGACCTCGATCACGC

### Statistical analysis

Datasets with only two independent groups were analysed for statistical significance using Student’s t test. Datasets with more than two groups were analysed using a one-way ANOVA. A value of P<0.05 was considered significant. Results are shown as means and standard deviations (SD)

## Results

### Genomic analysis of BP0414

BP0414 gene is described as a putative Mg^2+^ transporter of 519 bp which encodes a 172 amino acid protein with a theoretical molecular mass of 18 kDa. The amino acid sequence is 23% identical to the MgtC of *Salmonella enterica* serovar Typhimurium, 23% identical to the MgtC of *Burkholderia cenocepacia*, and 21% identical to the MgtC of *Mycobacterium tuberculosis*. Importantly, BP0414 contains the conserved hydrophobic N-terminal “MgtC domain” (S3 Fig). BP0414 is located between a pseudogene (BP0412) and *terC* gene (BP0415) encoding a putative membrane protein involved in tellurite resistance (S4 Fig). This genomic context suggests that, as found for *Burkholderia cenocepacia* MgtC [27], *B*. *pertussis* MgtC is not part of an operon. Indeed, data from a recently established primary transcriptome of *B*. *pertussis* [28] shows that BP0414 is expressed from its own promoter as a monocistronic transcript.

### Intracellular *B*. *pertussis* expresses MgtC

**Fig 1:**
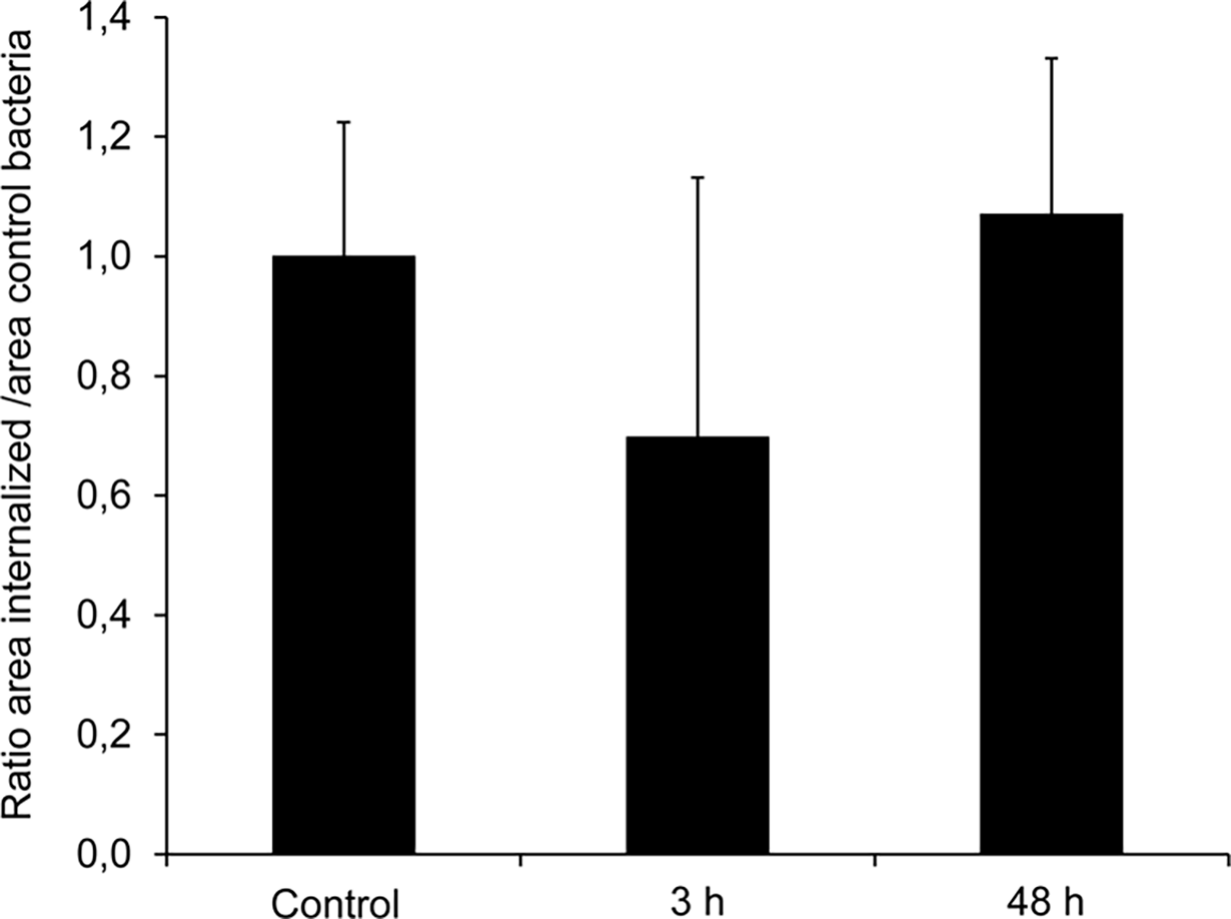
Single Reaction Monitoring (SRM) of BP0414 (MgtC). Relative abundance of BP0414 in extracellular bacteria (control), intracellular bacteria recovered 3 h post-infection (3 h), and intracellular bacteria recovered 48 h post-infection (48 h) are shown. Two independent biological replicates with two technical replicates each were analyzed. Values are expressed as ratios relative to the control. Data represent mean relative protein levels ± SD.

In a previous study we analyzed the evolution of the *B*. *pertussis* proteome during the intracellular infection [8]. We found the MgtC homolog (BP0414) expressed in the intracellular bacteria both at 3 and 48 h after infection of THP-1 cells (PRIDE Identifier PXD002997). In order to confirm the proteomics results, we used the SRM method [29] to search for MgtC in the infecting (extracellular) and the intracellular bacteria at 3 and 48 hours after THP-1 infection. MgtC expression was confirmed in all the bacterial samples (S5 Fig). Fig 1 shows that the abundance of this protein remained unchanged in the intracellular bacteria over the time of intracellular infection.

### MgtC is involved in *B*. *pertussis* intracellular survival

**Fig 2:**
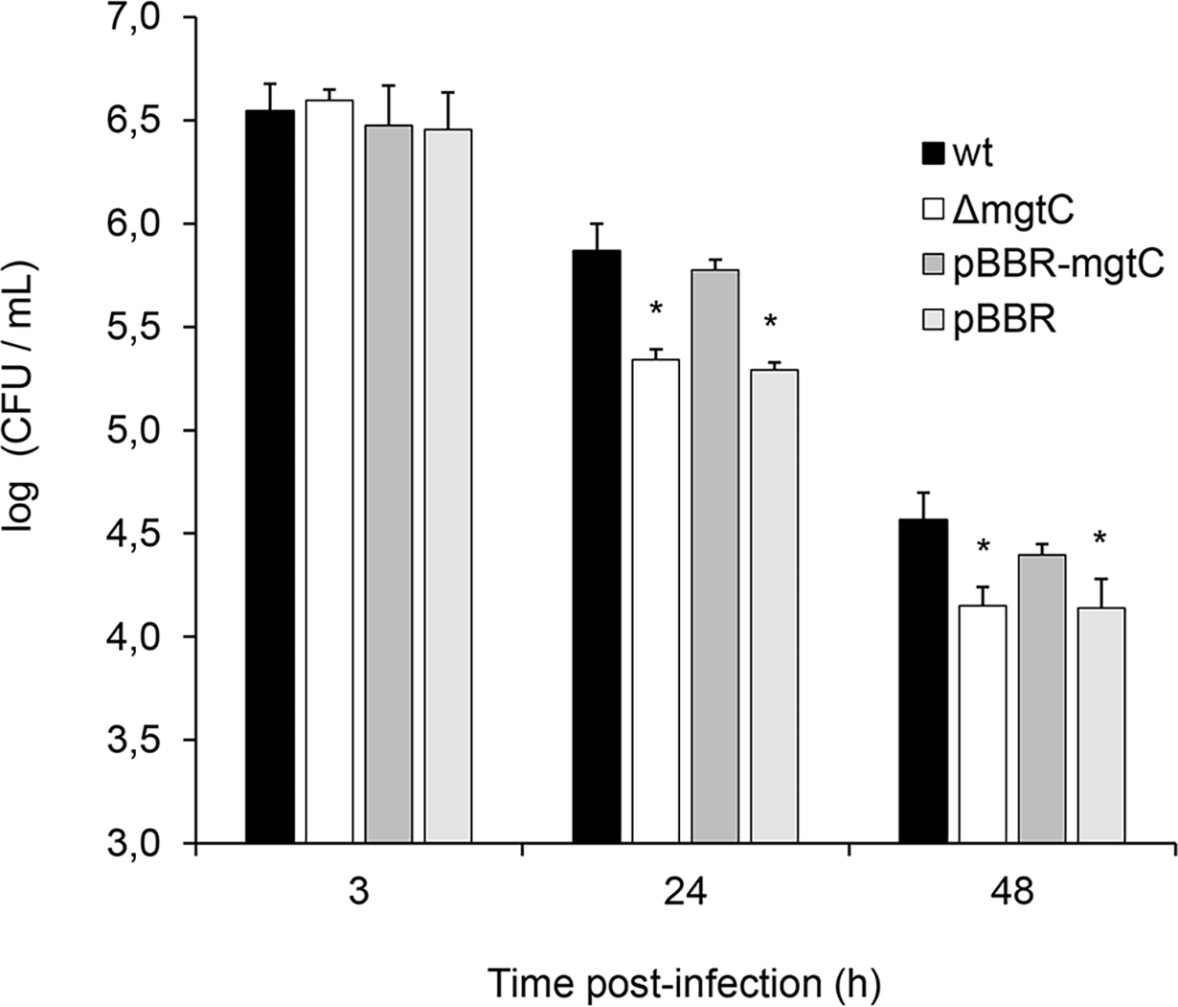
MgtC is required for intracellular survival. Wild type *B*. *pertussis* (wt), *B*. *pertussis mgtC* deficient mutant (Δ*mgtC*), *B*. *pertussis mgtC* deficient mutant carrying the pBBR1MCS vector (pBBR), and *B*. *pertussis mgtC* deficient mutant carrying a pBBR1MCS derivative that encodes *mgtC* (pBBR-*mgtC*) were incubated with THP-1 macrophages (MOI: 100) for 2 h at 37°C, washed, and further incubated with polymyxin B to kill the extracellular bacteria. The number of CFU was determined at 3, 24, and 48 hours post-infection. The means ± SD of triplicates of one representative experiment out of three performed are given. The number of CFU per mL of Δ*mgtC* and pBBR at 24 and 48 h post-infection was significantly different from the number of CFU per mL of the wt and pBBR-*mgtC* strains at the respective time post-infection. Statistical significance analysis was performed using one-way ANOVA (*P<0.05).

**Fig 3:**
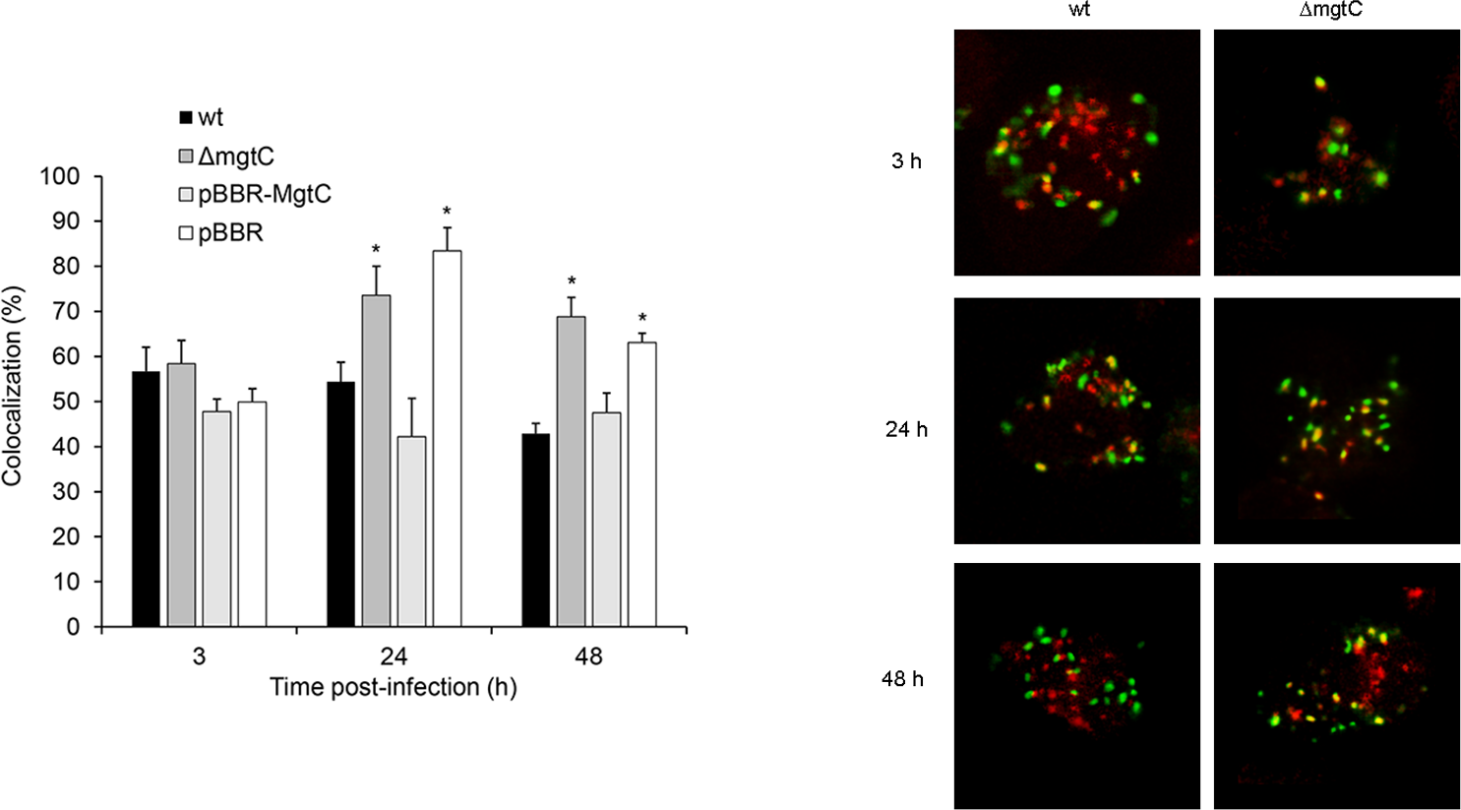
Time course of *B*. *pertussis* colocalization with the acidotropic dye LysoTracker. Wild type *B*. *pertussis* (wt), *B*. *pertussis mgtC* deficient mutant (Δ*mgtC*), *B*. *pertussis mgtC* deficient mutant carrying the pBBR1MCS vector (pBBR), and *B*. *pertussis mgtC* deficient mutant carrying a pBBR1MCS derivative that encodes *mgtC* (pBBR-*mgtC*) were incubated with THP-1 cells (MOI: 100) for 2 h at 37°C. After being washed, the macrophages were incubated with polymyxin B (100 μg/mL) prior incubation with LysoTracker (3 h), or further incubated with RPMI-FBS-polymyxin B (5 μg/mL) for other 24 and 48 h before treatment with the acidotropic dye LysoTracker (red). Intracellular bacteria were green fluorescently labelled prior to confocal microscopy analysis. Colocalization of bacteria and LysoTracker is refected by yellow areas. The bars indicate the percentage of LysoTracker-positive phagosomes. The means ± SD of triplicates of one representative experiment out of two performed are given. The percentage of Δ*mgtC* and pBBR colocalizing with LysoTracker at 24 h and 48 h post-infection was significantly different from the number of wt and pBBR-*mgtC* bacteria colocalizing with LysoTracker at the respective time points. Statistical significance analysis was performed using one-way ANOVA (*P<0.05). Confocal microscopy images of a representative experiment are shown.

In order to evaluate whether MgtC is involved in the intracellular survival of *B*. *pertussis* a mutant strain deficient in *mgtC* expression (Δ*mgtC*) was constructed by homologous recombination and the survival of the wild type (wt) and the Δ*mgtC* strains inside THP-1 macrophages was compared. THP-1 macrophages were infected with each strain, and the intracellular bacteria were recovered at 3 h, 24 h and 48 h post-infection. Microscopic studies showed that there were no significant differences in the level of adhesion to or phagocytosis by THP-1 cells between the strains (S6 Fig). Additionally, both strains showed similar survival 3 hours after infection, suggesting that MgtC is neither involved in bacterial phagocytosis nor in the early events after internalization (Fig 2). Conversely, at 24 h and 48 h post-infection less viable intracellular Δ*mgtC* bacteria were recovered as compared with the wt strain, suggesting the involvement of MgtC in the intracellular survival (Fig 2). The Δ*mgtC* strain survival defect was completely restored by complementation with a plasmid harbouring the *mgtC* gene (Fig 2), confirming that the decreased intracellular survival of the MgtC defective mutant was caused by the lack of this gene rather than an eventual polar effect caused by the mutation. Accordingly, Fig 3 shows that the lack of *mgtC* expression led to a higher proportion of bacteria trafficked to lysosomes both at 24 and 48 h post-infection, as determined by the colocalization of intracellular bacteria with the acidotrophic dye LysoTracker. Control studies showed that the MgtC deficient mutant had no growth defect in standard *in vitro* growth conditions using SS medium as compared with the wild type. Taken together, these results indicate that MgtC plays a role in *B*. *pertussis* survival within macrophages.

### MgtC is required for acidic tolerance

**Fig 4:**
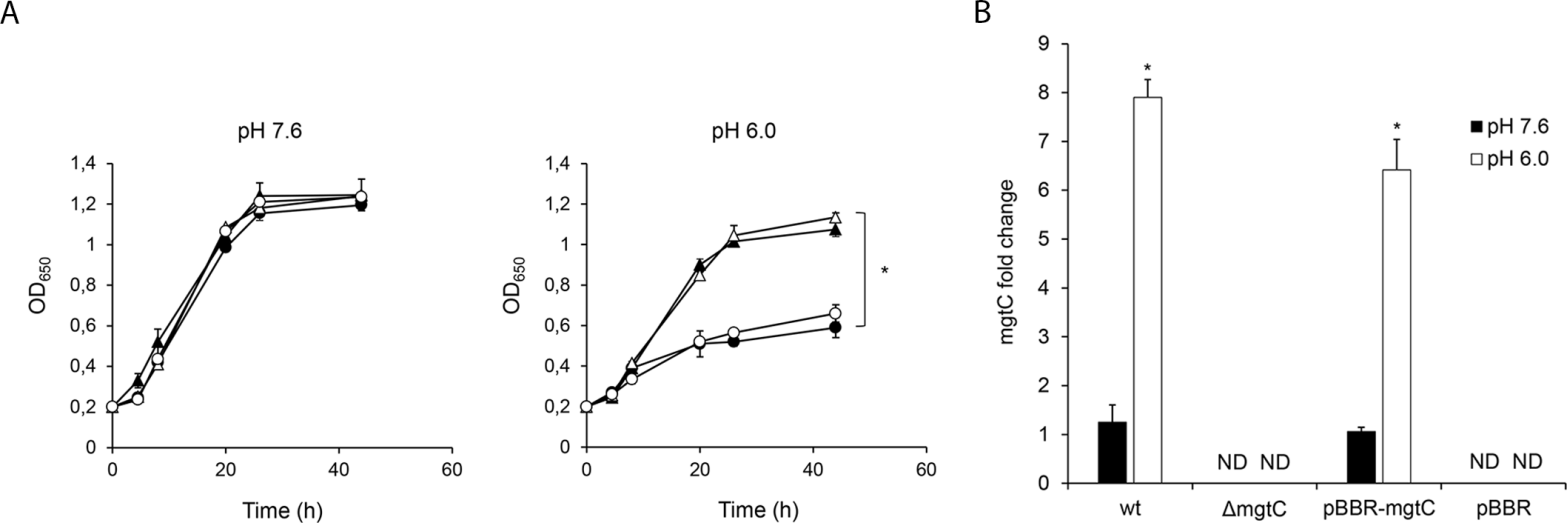
MgtC is required for growth in acid environments. (A) Time course of wild type *B*. *pertussis* (wt, filled triangles), *B*. *pertussis mgtC* deficient mutant (Δ*mgtC*, filled circles), *B*. *pertussis mgtC* deficient mutant carrying the pBBR1MCS vector (pBBR, open circles), and *B*. *pertussis mgtC* deficient mutant carrying a pBBR1MCS derivative that encodes *mgtC* (pBBR-*mgtC*, open triangles) growth in SS-MES medium buffered at pH 7.6 or 6.0. The means ± SD of duplicates of one representative experiment out of two performed are given. The maximum OD650 of the wt and pBBR-*mgtC* strains grown at pH 6.0 were significantly different from the maximum OD650 of the Δ*mgtC* and pBBR strains. Statistical significance analysis was performed using one-way ANOVA (*P<0.05). (B) The expression level of *mgtC* of the wt, Δ*mgtC*, pBBR and pBBR-*mgtC* strains growing in the SS-MES medium buffered at pH 7.6 or 6.0 was measured by RT-qPCR. The *recA* gene was used as a housekeeping gene for normalization. The relative expression level of *mgtC* was calculated by the 2^−ΔΔCt^ method. The means ± SD of duplicates of one representative experiment out of two performed are given. The level of expression of *mgtC* in wild type *B*. *pertussis* growing in SS-MES medium buffered at pH 6.0 was significantly different from the level of expression of *mgtC* in wild type *B*. *pertussis* growing in SS-MES buffered at pH 7.6. Statistical significance analysis was performed using Student's t-test (*P<0.05). The level of expression of *mgtC* in *B*. *pertussis* pBBR-*mgtC* growing in SS-MES medium buffered at pH 6.0 was significantly different from the level of expression of *mgtC* in *B*. *pertussis* pBBR-*mgtC* growing in SS-MES buffered at pH 7.6. Statistical significance analysis was performed using Student's t-test (*P<0.05). ND: not detected.

Some members of the MgtC family have been found to be implicated in acid resistance [30]. Previous studies indicated that intracellular *B*. *pertussis* remains alive in compartments with early endosomal characteristics. The pH of the microenvironment in such compartments is around 6.0 which is usually detrimental for bacterial viability. Intracellular pathogens have developed mechanisms to avoid or resist the acidic environment of the endocytic organelles [31]. In order to investigate whether *B*. *pertussis* MgtC is involved in bacterial growth in acidic environments wt and Δ*mgtC* strains were grown in parallel under different pH controlled culture conditions (pH 7.6 and 6.0). No differences were observed in the growth of the different strains in culture media with controlled pH at 7.6 (Fig 4A). However, when compared to the wild type strain, the Δ*mgtC* strain showed a significant growth defect in the medium adjusted to pH 6.0, (Fig 4A). Accordingly, we found an up regulation of *mgtC* expression in the wild type strain growing at pH 6.0, as compared to the same strain growing at pH 7.6 (Fig 4B) thereby supporting the involvement of MgtC in the bacterial adaptation to mildly acidic conditions.

### MgtC is involved in *B*. *pertussis* growth under Mg^2+^ restricted conditions

**Fig 5:**
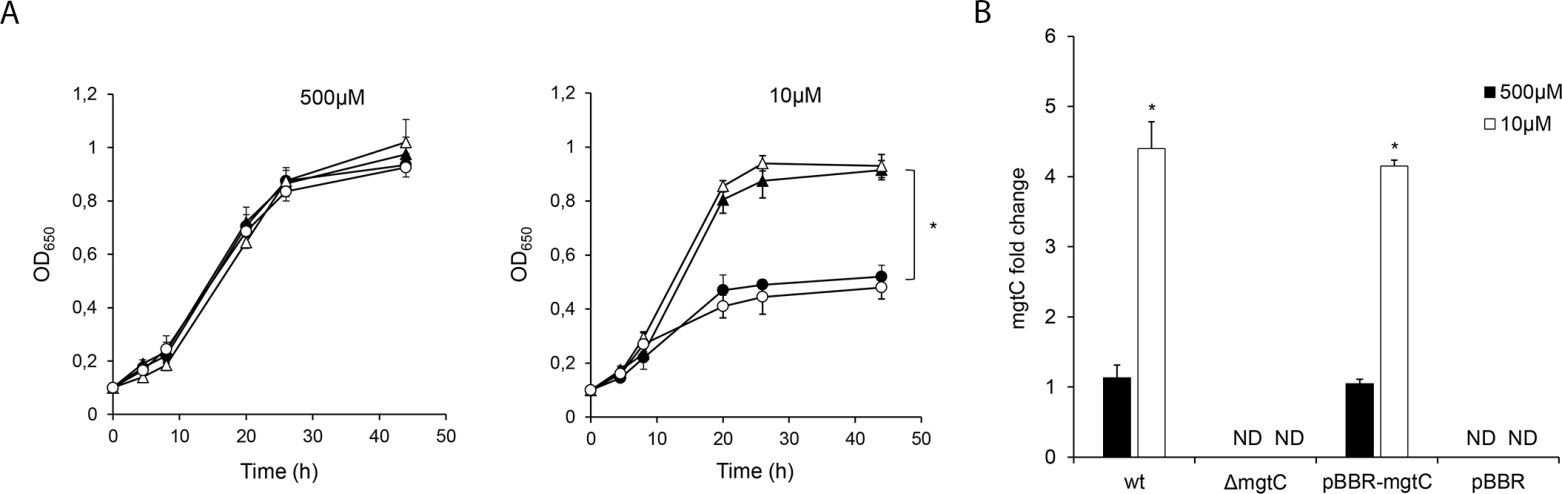
MgtC is required for growth under low magnesium availability. **(**A) Time course of wild type *B*. *pertussis* (wt, filled triangles), *B*. *pertussis mgtC* deficient mutant (Δ*mgtC*, filled circles), *B*. *pertussis mgtC* deficient mutant carrying the pBBR1MCS vector (pBBR, open circles), and *B*. *pertussis mgtC* deficient mutant carrying a pBBR1MCS derivative that encodes *mgtC* (pBBR-*mgtC*, open triangles) growth in SS medium containing 500 μM (SS) or 10 μM (SS-Mg) of MgCl_2_. The means ± SD of duplicates of one representative experiment out of two performed are given. The maximum OD650 of the wt and pBBR-*mgtC* strains grown under Mg^2+^-depleted conditions were significantly different from the maximum OD650 of the Δ*mgtC* and pBBR strains grown under Mg^2+^-depleted conditions. Statistical significance analysis was performed using one-way ANOVA (*P<0.05). (B) The expression level of *mgtC* of the wt, Δ*mgtC*, pBBR and pBBR-*mgtC* strains growing in SS or SS-Mg was measured by RT-qPCR. The *recA* gene was used as a housekeeping gene for normalization. The relative expression level of each gene tested was calculated by the 2^-ΔΔCt^ method. The means ± SD of duplicates of one representative experiment out of two performed are given. The level of expression of *mgtC* in wild type *B*. *pertussis* growing in SS-Mg medium was significantly different from the level of expression of *mgtC* in wild type *B*. *pertussis* growing in SS medium. Statistical significance analysis was performed using Student's t-test (*P<0.05). The level of expression of *mgtC* in *B*. *pertussis* pBBR-*mgtC* growing in SS-Mg medium was significantly different from the level of expression of *mgtC* in *B*. *pertussis* pBBR-*mgtC* growing in SS medium. Statistical significance analysis was performed using Student's t-test (*P<0.05). ND: not detected.

Several studies indicate that MgtC promotes growth in magnesium depleted environments [9]. In order to evaluate whether MgtC has a role in *B*. *pertussis* growth under low magnesium availability, the ability of the wt and the Δ*mgtC* strains to grow under Mg^2+^-depleted and Mg^2+^-replete conditions was compared. While no difference was detected in the growth of the wt and Δ*mgtC* strains under Mg^2+^-replete conditions, the Δ*mgtC* strain displayed impaired growth when compared to the wild type strain under low Mg^2+^ availability suggesting that MgtC is required for *B*. *pertussis* growth under this condition (Fig 5A).

The transcriptional level of *mgtC* in *B*. *pertussis* growing under Mg^2+^-replete and Mg^2+^-depleted conditions was analysed by quantitative RT-PCR. Fig 4B shows that *mgtC* transcriptional level was induced by magnesium starvation indicating that, as found for other pathogens [18], the transcription of this gene is regulated by Mg^2+^ availability.

### Treatment of macrophages with bafilomycin A1 rescues the survival defect of the Δ*mgtC* strain

**Fig 6:**
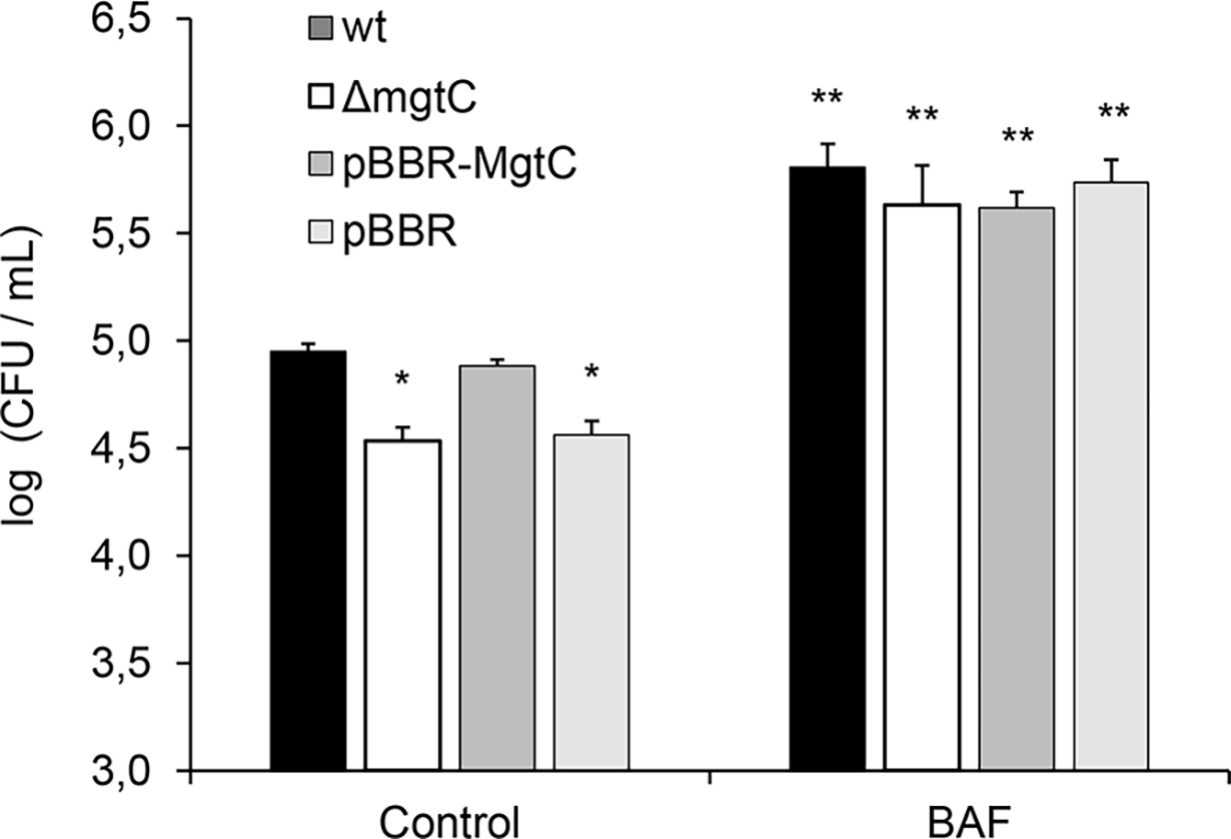
Treatment of THP-1 cells with bafilomycin A1 restores the intracellular survival defect of *B*. *pertussis mgtC* deletion mutant strain to wild type *B*. *pertussis* levels. THP-1 macrophages were treated with 50 nM bafilomycin A1 (BAF) dissolved in DMSO or with DMSO alone (control condition) for 1 hour prior infection with either wild type *B*. *pertussis* (wt), *B*. *pertussis mgtC* deficient mutant (Δ*mgtC*), *B*. *pertussis mgtC* deficient mutant carrying the pBBR1MCS vector (pBBR), and *B*. *pertussis mgtC* deficient mutant carrying a pBBR1MCS derivative that encodes *mgtC* (pBBR-*mgtC*). After 2 h of infection cells were treated with polymyxin B and 48 hours after infection intracellular survival was assessed by lysing cells and enumerating CFUs. The means ± SD of triplicates of one representative experiment out of two performed are given. The survival of the Δ*mgtC* and pBBR strains in control conditions was significantly different from the survival of the wt and pBBR-*mgtC* strains in control conditions. Statistical significance analysis was performed using one-way ANOVA (*P<0.05). The survival of wt, Δ*mgtC*, pBBR, and pBBR-*mgtC* strains in BAF treated cells was significantly higher than the survival of any of these strains in untreated cells (control conditions). Statistical significance analysis was performed using one-way ANOVA (**P<0.05).

In order to gain insight into the role of MgtC in the survival of intracellular bacteria we next investigated whether it is involved in bacterial adaptation to the acidic environmental conditions found inside the phagosome. To this end, THP-1 cells were incubated with or without BAF, an inhibitor of vacuolar ATPase that blocks the acidification of intracellular compartments [21] and the intracellular survival of both strains in treated and non-treated THP-1 cells was compared 48 hours post-infection. The number of viable intracellular wt and Δ*mgtC* bacteria significantly increased in BAF-treated macrophages. Notably, in THP-1 cells treated with bafilomycin A1 the survival of the *mgtC* deletion mutant was similar to that of the wt strain (Fig 6). These results suggest that MgtC is primarily involved in the adaptation of *B*. *pertussis* to the acidic conditions found inside the phagosome.

## Discussion

Several studies showed that *B*. *pertussis* is able to persist inside the host cells and thereby evade the host defence mechanisms [2–4]. In a recent study [8], in which the changes in *B*. *pertussis* proteome during macrophage infection were investigated, we identified a protein which was not studied before in *Bordetella* and shares homology with MgtC, a factor that was proved to be relevant for intracellular survival in other pathogens. The proteomic data were further confirmed by SRM, an established clinical chemistry tool for the quantification of proteins in complex biological samples [32, 33]. By means of this technique we confirmed the production of MgtC by *B*. *pertussis* in the intracellular bacteria.

Previous studies have shown that MgtC promotes the survival of several pathogens inside the macrophages by supporting bacterial adaptation to the intracellular environment [9, 27]. In this study, we found that MgtC is also required for *B*. *pertussis* to establish a successful infection of macrophages as the mutant strain lacking the MgtC showed a decrease in the intracellular survival as compared with the parental strain. Previous trafficking studies indicate that during the first hours following phagocytosis, a high percentage of *B*. *pertussis* bacteria were trafficked to acidic compartments [3]. However, roughly one-fourth of the bacteria taken up by the macrophages evade this initial killing event and remain alive in mildly acidic compartments with characteristics of early endosomes [3]. This non-bactericidal trafficking showed to be dependent on the viability of the bacteria, as heat killed *B*. *pertussis* was trafficked exclusively to lysosomes. This study revealed an increased colocalization of the a *B*. *pertussis* mutant strain lacking MgtC with lysosomes as compared with the wild type strain indicating that MgtC might be involved in *B*. *pertussis* survival inside macrophages.

Several pathogens require MgtC to grow in Mg^2+^-deprived environments [18] or under acidic conditions [30]. Likewise, using standard *in vitro* growth conditions we found that *B*. *pertussis* MgtC plays a dual role by promoting growth under both mildly acidic conditions and low Mg^2+^ availability. This dual role might be explained by the reported activity of MgtC in other pathogens, namely, the inhibition of the bacterial ATP synthase in order to reduce the ATP production [12]. In Gram-negative bacteria, ATP synthases are located in the inner membrane and use the chemiosmotic proton gradient to power ATP synthesis. The bacterial outer membrane is permeable to protons, which means that a change in the environmental pH determines a change in the proton concentration in the periplasm. In order to avoid the production of supra-physiological levels of ATP at low pH, MgtC is usually overproduced to modulate the activity of the ATP synthase [12]. Additionally, a decrease in ATP synthase activity leads to a reduction of the level of cytoplasmic ATP, a strong magnesium chelator, with the concomitant increase in Mg^2+^ bioavailability [34]. We show that under magnesium limitation *mgtC* expression is increased suggesting that, as described for other pathogens, *B*. *pertussis* MgtC might be involved in fine-tuning the bacterial ATP and magnesium concentration.

*B*. *pertussis*-containing phagosome is still not fully characterized. Previous studies have indicated that this pathogen might reside in compartments with early endosomal characteristics [3]. Endosomal vesicles have mildly acidic pH, with an average value of 6.0 [31], a pH at which *B*. *pertussis* growth showed to be dependent on MgtC according to our results. The magnesium concentration inside these compartments, on the other hand, is still controversial [35, 36]. In order to evaluate the contribution of the pH and the Mg^2+^ concentration to the impaired survival of the *mgtC* deletion mutant inside the cells, we treated the human macrophages with bafilomycin A1, a drug that prevents phagosomal acidification [21]. The drug not only increased the survival of the wild type strain, as expected, but also suppressed the survival defect of the Δ*mgtC* strain. According to our results *B*. *pertussis* requires MgtC to overcome the low Mg^2+^ availability even in neutral environments (Fig 5B). This finding, together with the similar intracellular viability observed for the *mgtC* defective mutant and the wild type strain in the presence of Bafilomicyn A, indicate that intracellular *B*. *pertussis* is not Mg^2+^-depleted, which is in agreement with several studies showing that phagosomes are usually not deprived of this cation [35, 36]. Altogether, these results suggest that the main role of MgtC in intracellular *B*. *pertussis* might be related to the bacterial survival in the mildly acidic environment found inside the phagosome.

A homolog of *B*. *pertussis* MgtC, BPP3813, is also present in *B*. *parapertussis*, a closely related human pathogen that was also found to persist within human macrophages [3, 37]. *B*. *pertussis* and *B*. *parapertussis* evolved from a common ancestor, *Bordetella bronchiseptica*, in a process that involved large-scale gene loss and inactivation [38]. The fact that MgtC was conserved with almost complete sequence identity despite genome reduction suggests that this protein plays a role in their pathogenesis.

The results obtained in this study demonstrate that *B*. *pertussis* MgtC promotes the bacterial intracellular survival, which seems to be linked to its role in the bacterial adaptation to the acidic conditions. As found for other pathogens, this study identifies *B*. *pertussis* MgtC as a virulence factor associated with intramacrophage survival, adding a new layer of complexity to this interaction and underlying the relevance of an intracellular phase during *B*. *pertussis* infection.

## Supporting information

S1 Fig Nucleotide sequence of the *mgtC* (BP0414) genomic region in the wild type and the Δ*mgtC* mutant strains of *B*. *pertussis*. Nucleotide sequence of the *mgtC* genomic of the wild-type strain (wt) and the *mgtC* mutant strain (Δ*mgtC*) were aligned. The *mgtC* DNA coding sequence is highlighted in red and the *Spe*I site used for construction of the mutant strain is underlined.
(TIF)

S2 Fig Effect of bafilomycin A1 in THP-1 macrophages. THP-1 cells were differentiated into macrophages with PMA and further incubated with 50 nM of bafilomycin A1 dissolved in DMSO to neutralize the vacuolar pH or treated with DMSO alone (control) for 1 h or 48 h. Cells were incubated with LysoTracker for acidic organelle staining and processed for confocal microscopy. Representative confocal microscopy images of both incubation times are shown.
(TIF)

S3 Fig Sequence alignment analysis of characterized MgtC proteins of different pathogens and *B*. *pertussis* MgtC. The alignment of amino acid sequences of MgtC proteins was performed using Clustal Omega. Conserved amino acids are shaded dark blue (>80% identity) while semi-conserved amino acids are shaded blue (>60% identity) or light blue (>40% identity). *B*. *pertussis* conserves several amino acids of the hydrophobic N-terminal “MgtC domain”. Protein sequences used for the alignment are from *Burkholderia cenocepacia* K56-2 (EPZ84702.1), *Bordetella pertussis* Tohama I (CAE44745.1), *Brucella suis* 1330 (KFJ26613.1), *Mycobacterium tuberculosis* Erdman (BAL65794.1), *Pseudomonas aeruginosa* PAO1 (NP_253325.1), *Salmonella* Typhimurium 14028s (AAD16960.1) and *Salmonella* Typhi STH2370 (ETZ13253.1).
(TIF)

S4 Fig Genetic organization of the region surrounding *mgtC* (BP0414) in *B*. *pertussis*. Open reading frames are depicted by arrows indicating the presumed direction of transcription. The *mgtC* gene appears to be a discrete transcriptional unit located downstream of a *fusA* pseudogene. Putative promoter and terminator of *mgtC* are indicated by a vertical arrow and a hairpin symbol, respectively.
(TIF)

S5 Fig Representative chromatograms of the transitions for the peptide LGNEGEIR acquired at different time points. Coloured traces refer to extracted ion chromatograms for the various product ions of the peptide LGNEGEIR.
(TIF)

S6 Fig MgtC is not involved in the adhesion or phagocytosis to THP-1 cells. (A) Wild type *B*. *pertussis* (wt), *B*. *pertussis mgtC* deficient mutant *(*Δ*mgtC)* and complemented *mgtC* deficient mutant strain (pBBR-*mgtC)* were incubated with THP-1 macrophages (MOI: 100) for 2 h at 37°C. After being washed, the cells were fixed and extracellular and intracellular bacteria were quantified by double immunofluorescence staining. The number of macrophage-associated bacteria was determined by fluorescence microscopy. At least 50 cells were counted per sample. The means ± SD of triplicates of one representative experiment out of three performed are given. No statistically significant differences were found in the number of bacteria associated to the THP-1 macrophages between the strains (B) *B*. *pertussis* (wt), *B*. *pertussis mgtC* deficient mutant (Δ*mgtC*) and complemented *mgtC* deficient mutant strain (pBBR-*mgtC)* were incubated with THP-1 macrophages (MOI: 100) for 2 h at 37°C. After being washed, the cells were fixed and extracellular and intracellular bacteria were quantified by double immunofluorescence staining. The numbers of phagocytosed bacteria were determined by fluorescence microscopy. At least 50 cells were counted per sample. Phagocytosis was expressed as the percentage of associated bacteria that were internalized. The means ± SD of triplicates of one representative experiment out of three performed are given. No statistically significant differences were found in the level of bacterial phagocytosis by THP-1 macrophages between the strains.
(TIF)

S1 Table Transition list for final data acquisition by SRM. The parent proteins, the peptide sequences, the mass/charge ratios of the precursor and product ions, and the collision energies applied are listed.
(XLSX)

S1 Data Raw data of Fig 2. (XLSX)
