# Supplementary figures and images for: A Bordetella pertussis MgtC homolog plays a role in the intracellular survival
Source: PLoS One. 2018 Aug 30;13(8):e0203204. doi: 10.1371/journal.pone.0203204 (PMC6117051; doi:10.1371/journal.pone.0203204)

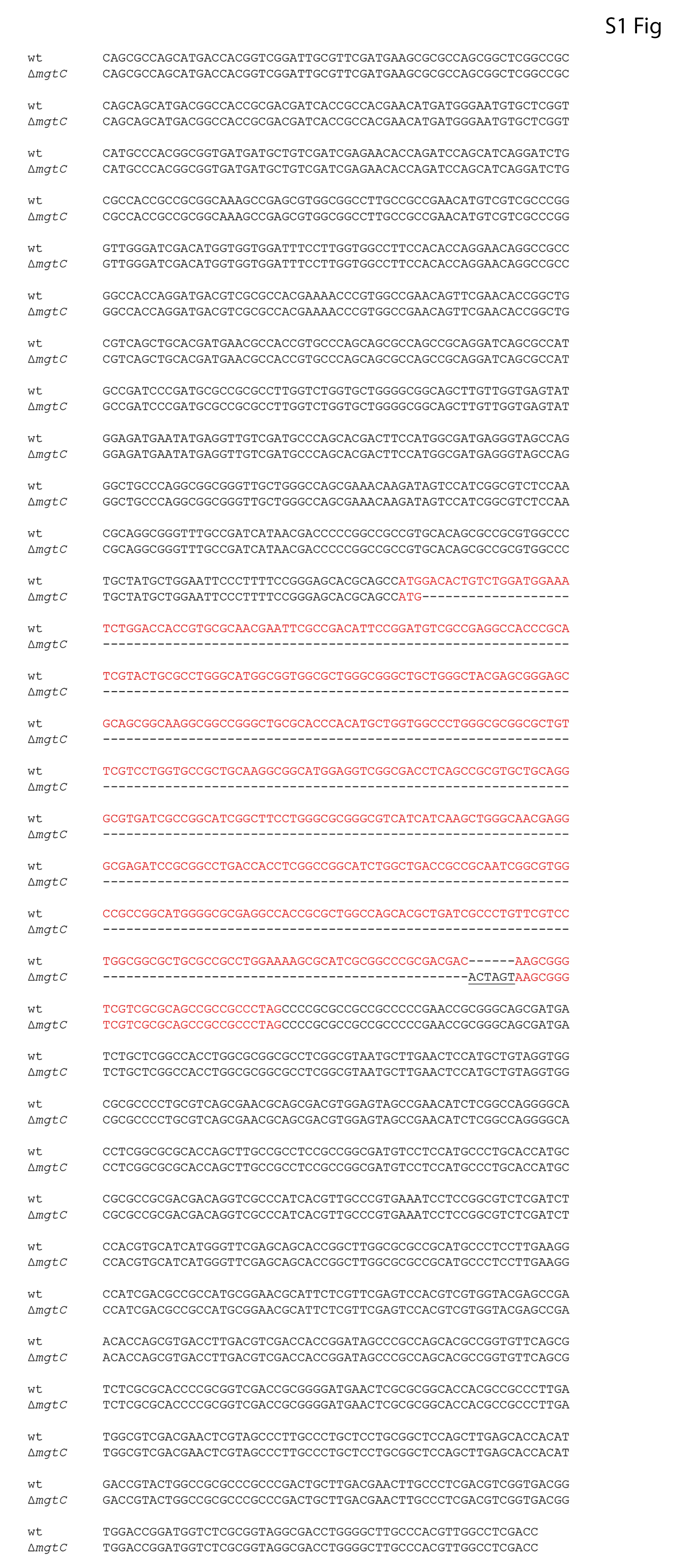

Supplement: S1 Fig — Nucleotide sequence of the mgtC genomic of the wild-type strain (wt) and the mgtC mutant strain (ΔmgtC) were aligned. The mgtC DNA coding sequence is highlighted in red and the SpeI site used for construction of the mutant strain is underlined. (TIF) [file pone.0203204.s001.tif]

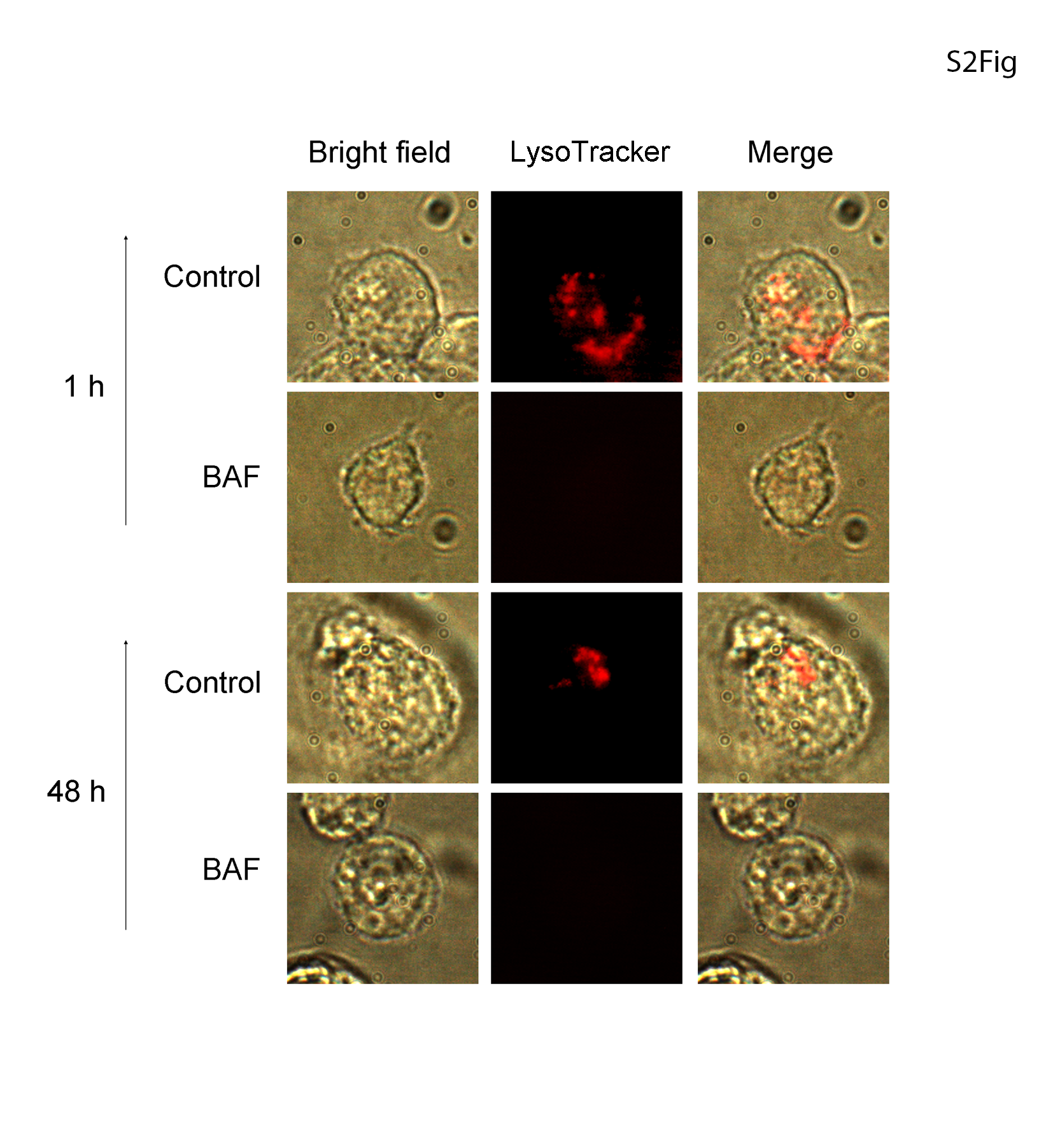

Supplement: S2 Fig — THP-1 cells were differentiated into macrophages with PMA and further incubated with 50 nM of bafilomycin A1 dissolved in DMSO to neutralize the vacuolar pH or treated with DMSO alone (control) for 1 h or 48 h. Cells were incubated with LysoTracker for acidic organelle staining and processed for confocal microscopy. Representative confocal microscopy images of both incubation times are shown. (TIF) [file pone.0203204.s002.tif]

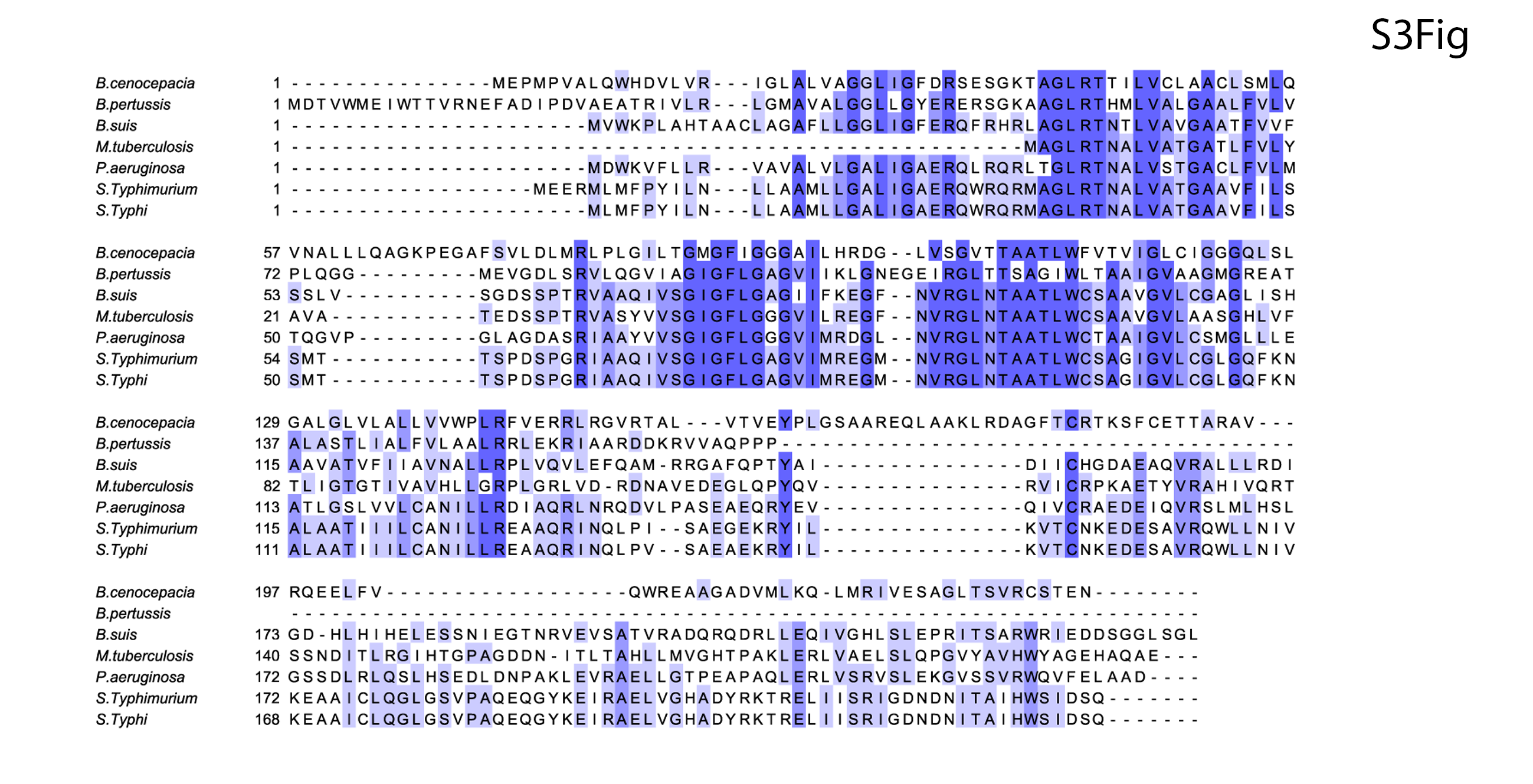

Supplement: S3 Fig — The alignment of amino acid sequences of MgtC proteins was performed using Clustal Omega. Conserved amino acids are shaded dark blue (>80% identity) while semi-conserved amino acids are shaded blue (>60% identity) or light blue (>40% identity). B. pertussis conserves several amino acids of the hydrophobic N-terminal “MgtC domain”. Protein sequences used for the alignment are from Burkholderia cenocepacia K56-2 (EPZ84702.1), Bordetella pertussis Tohama I (CAE44745.1), Brucella suis 1330 (KFJ26613.1), Mycobacterium tuberculosis Erdman (BAL65794.1), Pseudomonas aeruginosa PAO1 (NP_253325.1), Salmonella Typhimurium 14028s (AAD16960.1) and Salmonella Typhi STH2370 (ETZ13253.1). (TIF) [file pone.0203204.s003.tif]

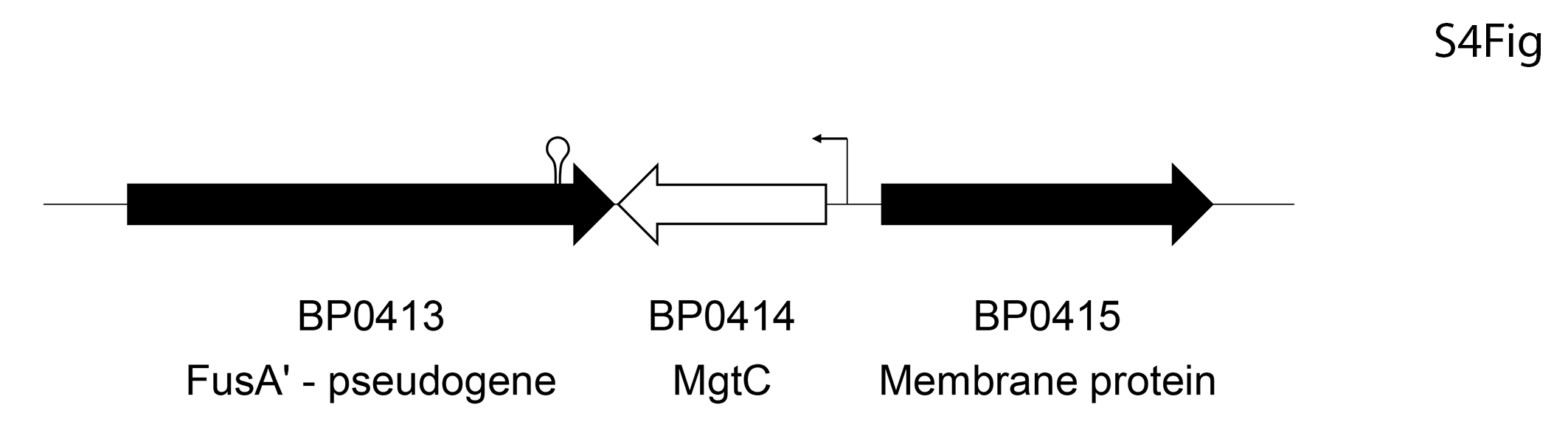

Supplement: S4 Fig — Open reading frames are depicted by arrows indicating the presumed direction of transcription. The mgtC gene appears to be a discrete transcriptional unit located downstream of a fusA pseudogene. Putative promoter and terminator of mgtC are indicated by a vertical arrow and a hairpin symbol, respectively. (TIF) [file pone.0203204.s004.tif]

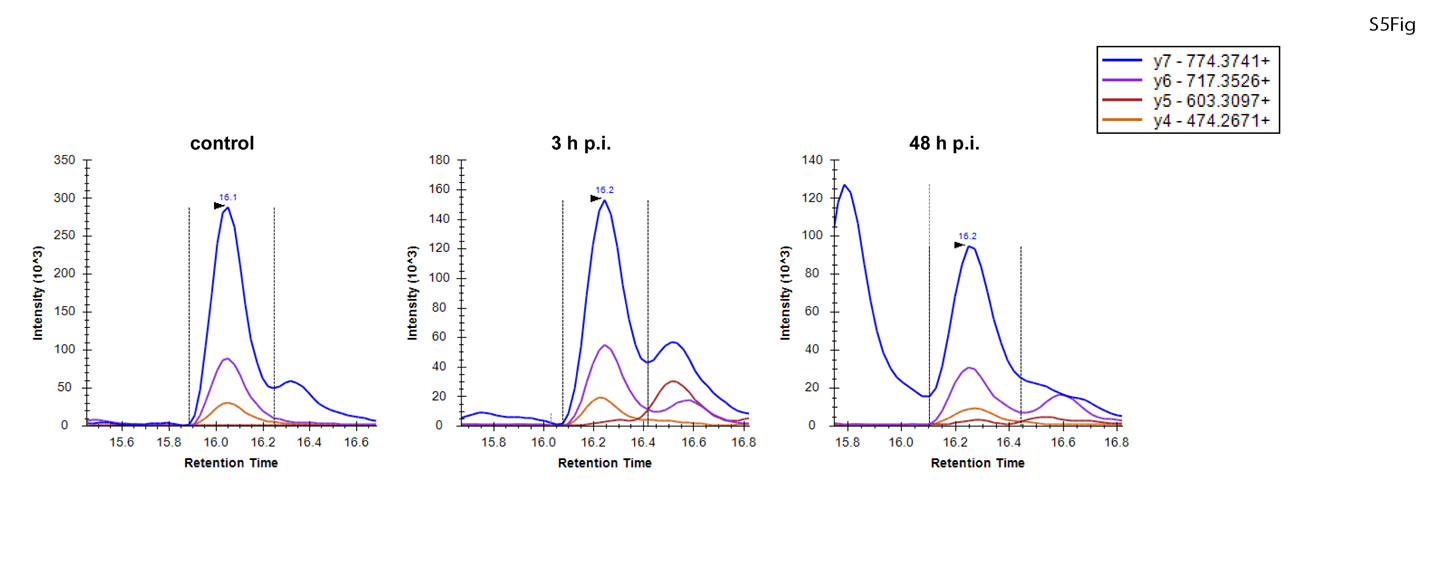

Supplement: S5 Fig — Coloured traces refer to extracted ion chromatograms for the various product ions of the peptide LGNEGEIR. (TIF) [file pone.0203204.s005.tif]

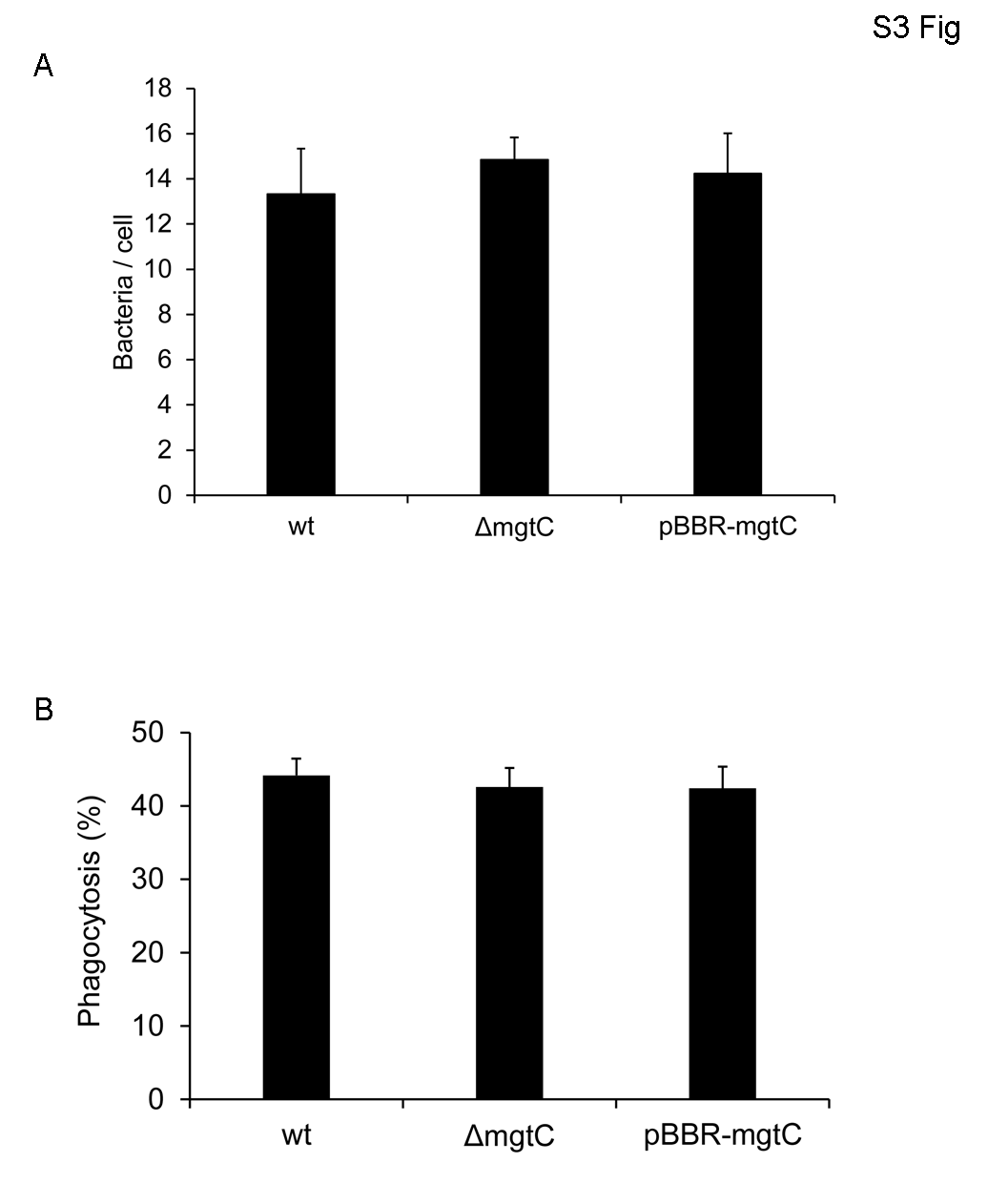

Supplement: S6 Fig — (A) Wild type B. pertussis (wt), B. pertussis mgtC deficient mutant (ΔmgtC) and complemented mgtC deficient mutant strain (pBBR-mgtC) were incubated with THP-1 macrophages (MOI: 100) for 2 h at 37°C. After being washed, the cells were fixed and extracellular and intracellular bacteria were quantified by double immunofluorescence staining. The number of macrophage-associated bacteria was determined by fluorescence microscopy. At least 50 cells were counted per sample. The means ± SD of triplicates of one representative experiment out of three performed are given. No statistically significant differences were found in the number of bacteria associated to the THP-1 macrophages between the strains (B) B. pertussis (wt), B. pertussis mgtC deficient mutant (ΔmgtC) and complemented mgtC deficient mutant strain (pBBR-mgtC) were incubated with THP-1 macrophages (MOI: 100) for 2 h at 37°C. After being washed, the cells were fixed and extracellular and intracellular bacteria were quantified by double immunofluorescence staining. The numbers of phagocytosed bacteria were determined by fluorescence microscopy. At least 50 cells were counted per sample. Phagocytosis was expressed as the percentage of associated bacteria that were internalized. The means ± SD of triplicates of one representative experiment out of three performed are given. No statistically significant differences were found in the level of bacterial phagocytosis by THP-1 macrophages between the strains. (TIF) [file pone.0203204.s006.tif]
